# Supplementary material for: Using WGCNA (weighted gene co-expression network analysis) to identify the hub genes of skin hair follicle development in fetus stage of Inner Mongolia cashmere goat
Source: PLoS One. 2020 Dec 22;15(12):e0243507. doi: 10.1371/journal.pone.0243507 (PMC7755285; doi:10.1371/journal.pone.0243507)
Supplement: S1 File — (ZIP) [file pone.0243507.s005.zip › html/00Index.html]

R: Weighted Correlation Network Analysis

# Weighted Correlation Network Analysis

---

## Documentation for package ‘WGCNA’ version 1.69

- DESCRIPTION file.

## Help Pages

A
B
C
D
E
F
G
H
I
K
L
M
N
O
P
Q
R
S
T
U
V
W

## -- A --

|  |  |
| --- | --- |
| accuracyMeasures | Accuracy measures for a 2x2 confusion matrix or for vectors of predicted and observed values. |
| addBlockToBlockwiseData | Create, merge and expand BlockwiseData objects |
| addErrorBars | Add error bars to a barplot. |
| addGrid | Add grid lines to an existing plot. |
| addGuideLines | Add vertical "guide lines" to a dendrogram plot |
| addTraitToMEs | Add trait information to multi-set module eigengene structure |
| adjacency | Calculate network adjacency |
| adjacency.fromSimilarity | Calculate network adjacency |
| adjacency.polyReg | Adjacency matrix based on polynomial regression |
| adjacency.splineReg | Calculate network adjacency based on natural cubic spline regression |
| AFcorMI | Prediction of Weighted Mutual Information Adjacency Matrix by Correlation |
| alignExpr | Align expression data with given vector |
| allocateJobs | Divide tasks among workers |
| allowWGCNAThreads | Allow and disable multi-threading for certain WGCNA calculations |
| automaticNetworkScreening | One-step automatic network gene screening |
| automaticNetworkScreeningGS | One-step automatic network gene screening with external gene significance |

## -- B --

|  |  |
| --- | --- |
| BD.actualFileNames | Various basic operations on 'BlockwiseData' objects. |
| BD.blockLengths | Various basic operations on 'BlockwiseData' objects. |
| BD.checkAndDeleteFiles | Various basic operations on 'BlockwiseData' objects. |
| BD.getData | Various basic operations on 'BlockwiseData' objects. |
| BD.getMetaData | Various basic operations on 'BlockwiseData' objects. |
| BD.nBlocks | Various basic operations on 'BlockwiseData' objects. |
| bicor | Biweight Midcorrelation |
| bicorAndPvalue | Calculation of biweight midcorrelations and associated p-values |
| bicovWeightFactors | Weights used in biweight midcovariance |
| bicovWeights | Weights used in biweight midcovariance |
| bicovWeightsFromFactors | Weights used in biweight midcovariance |
| binarizeCategoricalColumns | Turn categorical columns into sets of binary indicators |
| binarizeCategoricalColumns.forPlots | Turn categorical columns into sets of binary indicators |
| binarizeCategoricalColumns.forRegression | Turn categorical columns into sets of binary indicators |
| binarizeCategoricalColumns.pairwise | Turn categorical columns into sets of binary indicators |
| binarizeCategoricalVariable | Turn a categorical variable into a set of binary indicators |
| BlockInformation | Create a list holding information about dividing data into blocks |
| blockSize | Attempt to calculate an appropriate block size to maximize efficiency of block-wise calcualtions. |
| blockwiseConsensusModules | Find consensus modules across several datasets. |
| BlockwiseData | Create, merge and expand BlockwiseData objects |
| blockwiseIndividualTOMs | Calculation of block-wise topological overlaps |
| blockwiseModules | Automatic network construction and module detection |
| BloodLists | Blood Cell Types with Corresponding Gene Markers |
| blueWhiteRed | Blue-white-red color sequence |
| BrainLists | Brain-Related Categories with Corresponding Gene Markers |
| BrainRegionMarkers | Gene Markers for Regions of the Human Brain |
| branchEigengeneDissim | Branch dissimilarity based on eigennodes (eigengenes). |
| branchEigengeneSimilarity | Branch dissimilarity based on eigennodes (eigengenes). |
| branchSplit | Branch split. |
| branchSplit.dissim | Branch split based on dissimilarity. |
| branchSplitFromStabilityLabels | Branch split (dissimilarity) statistics derived from labels determined from a stability study |
| branchSplitFromStabilityLabels.individualFraction | Branch split (dissimilarity) statistics derived from labels determined from a stability study |
| branchSplitFromStabilityLabels.prediction | Branch split (dissimilarity) statistics derived from labels determined from a stability study |

## -- C --

|  |  |
| --- | --- |
| checkAdjMat | Check adjacency matrix |
| checkSets | Check structure and retrieve sizes of a group of datasets. |
| checkSimilarity | Check adjacency matrix |
| chooseOneHubInEachModule | Chooses a single hub gene in each module |
| chooseTopHubInEachModule | Chooses the top hub gene in each module |
| clusterCoef | Clustering coefficient calculation |
| coClustering | Co-clustering measure of cluster preservation between two clusterings |
| coClustering.permutationTest | Permutation test for co-clustering |
| collapseRows | Select one representative row per group |
| collapseRowsUsingKME | Selects one representative row per group based on kME |
| collectGarbage | Iterative garbage collection. |
| colQuantileC | Fast colunm- and row-wise quantile of a matrix. |
| conformityBasedNetworkConcepts | Calculation of conformity-based network concepts. |
| conformityDecomposition | Conformity and module based decomposition of a network adjacency matrix. |
| consensusCalculation | Calculation of a (single) consenus with optional data calibration. |
| consensusDissTOMandTree | Consensus clustering based on topological overlap and hierarchical clustering |
| consensusKME | Calculate consensus kME (eigengene-based connectivities) across multiple data sets. |
| consensusMEDissimilarity | Consensus dissimilarity of module eigengenes. |
| ConsensusOptions | Create a list holding consensus calculation options. |
| consensusOrderMEs | Put close eigenvectors next to each other in several sets. |
| consensusProjectiveKMeans | Consensus projective K-means (pre-)clustering of expression data |
| consensusRepresentatives | Consensus selection of group representatives |
| consensusTOM | Consensus network (topological overlap). |
| ConsensusTree | Create a new consensus tree |
| consensusTreeInputs | Get all elementary inputs in a consensus tree |
| convertNumericColumnsToNumeric | Convert character columns that represent numbers to numeric |
| cor | Fast calculations of Pearson correlation. |
| cor1 | Fast calculations of Pearson correlation. |
| corAndPvalue | Calculation of correlations and associated p-values |
| corFast | Fast calculations of Pearson correlation. |
| corPredictionSuccess | Qunatification of success of gene screening |
| corPvalueFisher | Fisher's asymptotic p-value for correlation |
| corPvalueStudent | Student asymptotic p-value for correlation |
| CorrelationOptions | Creates a list of correlation options. |
| correlationPreservation | Preservation of eigengene correlations |
| coxRegressionResiduals | Deviance- and martingale residuals from a Cox regression model |
| cutreeStatic | Constant-height tree cut |
| cutreeStaticColor | Constant height tree cut using color labels |

## -- D --

|  |  |
| --- | --- |
| disableWGCNAThreads | Allow and disable multi-threading for certain WGCNA calculations |
| displayColors | Show colors used to label modules |
| dynamicMergeCut | Threshold for module merging |

## -- E --

|  |  |
| --- | --- |
| empiricalBayesLM | Empirical Bayes-moderated adjustment for unwanted covariates |
| enableWGCNAThreads | Allow and disable multi-threading for certain WGCNA calculations |
| exportNetworkToCytoscape | Export network to Cytoscape |
| exportNetworkToVisANT | Export network data in format readable by VisANT |

## -- F --

|  |  |
| --- | --- |
| factorizeNonNumericColumns | Turn non-numeric columns into factors |
| fixDataStructure | Put single-set data into a form useful for multiset calculations. |
| formatLabels | Break long character strings into multiple lines |
| fundamentalNetworkConcepts | Calculation of fundamental network concepts from an adjacency matrix. |

## -- G --

|  |  |
| --- | --- |
| GOenrichmentAnalysis | Calculation of GO enrichment (experimental) |
| goodGenes | Filter genes with too many missing entries |
| goodGenesMS | Filter genes with too many missing entries across multiple sets |
| goodSamples | Filter samples with too many missing entries |
| goodSamplesGenes | Iterative filtering of samples and genes with too many missing entries |
| goodSamplesGenesMS | Iterative filtering of samples and genes with too many missing entries across multiple data sets |
| goodSamplesMS | Filter samples with too many missing entries across multiple data sets |
| greenBlackRed | Green-black-red color sequence |
| greenWhiteRed | Green-white-red color sequence |
| GTOMdist | Generalized Topological Overlap Measure |

## -- H --

|  |  |
| --- | --- |
| hierarchicalBranchEigengeneDissim | Branch dissimilarity based on eigennodes (eigengenes). |
| hierarchicalConsensusCalculation | Hierarchical consensus calculation |
| hierarchicalConsensusKME | Calculation of measures of fuzzy module membership (KME) in hierarchical consensus modules |
| hierarchicalConsensusMEDissimilarity | Hierarchical consensus calculation of module eigengene dissimilarity |
| hierarchicalConsensusModules | Hierarchical consensus network construction and module identification |
| hierarchicalConsensusTOM | Calculation of hierarchical consensus topological overlap matrix |
| hierarchicalMergeCloseModules | Merge close (similar) hierarchical consensus modules |
| hubGeneSignificance | Hubgene significance |

## -- I --

|  |  |
| --- | --- |
| ImmunePathwayLists | Immune Pathways with Corresponding Gene Markers |
| imputeByModule | Impute missing data separately in each module |
| individualTOMs | Calculate individual correlation network matrices |
| initProgInd | Inline display of progress |
| intramodularConnectivity | Calculation of intramodular connectivity |
| intramodularConnectivity.fromExpr | Calculation of intramodular connectivity |
| isMultiData | Determine whether the supplied object is a valid multiData structure |

## -- K --

|  |  |
| --- | --- |
| keepCommonProbes | Keep probes that are shared among given data sets |
| kMEcomparisonScatterplot | Function to plot kME values between two comparable data sets. |

## -- L --

|  |  |
| --- | --- |
| labeledBarplot | Barplot with text or color labels. |
| labeledHeatmap | Produce a labeled heatmap plot |
| labeledHeatmap.multiPage | Labeled heatmap divided into several separate plots. |
| labelPoints | Label scatterplot points |
| labels2colors | Convert numerical labels to colors. |
| list2multiData | Convert a list to a multiData structure and vice-versa. |
| lowerTri2matrix | Reconstruct a symmetric matrix from a distance (lower-triangular) representation |

## -- M --

|  |  |
| --- | --- |
| matchLabels | Relabel module labels to best match the given reference labels |
| matrixToNetwork | Construct a network from a matrix |
| mergeBlockwiseData | Create, merge and expand BlockwiseData objects |
| mergeCloseModules | Merge close modules in gene expression data |
| metaAnalysis | Meta-analysis of binary and continuous variables |
| metaZfunction | Meta-analysis Z statistic |
| minWhichMin | Fast joint calculation of row- or column-wise minima and indices of minimum elements |
| moduleColor.getMEprefix | Get the prefix used to label module eigengenes. |
| moduleEigengenes | Calculate module eigengenes. |
| moduleMergeUsingKME | Merge modules and reassign genes using kME. |
| moduleNumber | Fixed-height cut of a dendrogram. |
| modulePreservation | Calculation of module preservation statistics |
| mtd.apply | Apply a function to each set in a multiData structure. |
| mtd.applyToSubset | Apply a function to each set in a multiData structure. |
| mtd.branchEigengeneDissim | Branch dissimilarity based on eigennodes (eigengenes). |
| mtd.colnames | Get and set column names in a multiData structure. |
| mtd.mapply | Apply a function to elements of given multiData structures. |
| mtd.rbindSelf | Turn a multiData structure into a single matrix or data frame. |
| mtd.setAttr | Set attributes on each component of a multiData structure |
| mtd.setColnames | Get and set column names in a multiData structure. |
| mtd.simplify | If possible, simplify a multiData structure to a 3-dimensional array. |
| mtd.subset | Subset rows and columns in a multiData structure |
| multiData | Create a multiData structure. |
| multiData.eigengeneSignificance | Eigengene significance across multiple sets |
| multiData2list | Convert a list to a multiData structure and vice-versa. |
| multiGrep | Analogs of grep(l) and (g)sub for multiple patterns and relacements |
| multiGrepl | Analogs of grep(l) and (g)sub for multiple patterns and relacements |
| multiGSub | Analogs of grep(l) and (g)sub for multiple patterns and relacements |
| multiIntersect | Union and intersection of multiple sets |
| multiSetMEs | Calculate module eigengenes. |
| multiSub | Analogs of grep(l) and (g)sub for multiple patterns and relacements |
| multiUnion | Union and intersection of multiple sets |
| mutualInfoAdjacency | Calculate weighted adjacency matrices based on mutual information |

## -- N --

|  |  |
| --- | --- |
| nearestCentroidPredictor | Nearest centroid predictor |
| nearestNeighborConnectivity | Connectivity to a constant number of nearest neighbors |
| nearestNeighborConnectivityMS | Connectivity to a constant number of nearest neighbors across multiple data sets |
| networkConcepts | Calculations of network concepts |
| NetworkOptions | Create a list of network construction arguments (options). |
| networkScreening | Identification of genes related to a trait |
| networkScreeningGS | Network gene screening with an external gene significance measure |
| newBlockInformation | Create a list holding information about dividing data into blocks |
| newBlockwiseData | Create, merge and expand BlockwiseData objects |
| newConsensusOptions | Create a list holding consensus calculation options. |
| newConsensusTree | Create a new consensus tree |
| newCorrelationOptions | Creates a list of correlation options. |
| newNetworkOptions | Create a list of network construction arguments (options). |
| normalizeLabels | Transform numerical labels into normal order. |
| nPresent | Number of present data entries. |
| nSets | Number of sets in a multi-set variable |
| numbers2colors | Color representation for a numeric variable |

## -- O --

|  |  |
| --- | --- |
| orderBranchesUsingHubGenes | Optimize dendrogram using branch swaps and reflections. |
| orderMEs | Put close eigenvectors next to each other |
| orderMEsByHierarchicalConsensus | Order module eigengenes by their hierarchical consensus similarity |
| overlapTable | Calculate overlap of modules |
| overlapTableUsingKME | Determines significant overlap between modules in two networks based on kME tables. |

## -- P --

|  |  |
| --- | --- |
| pickHardThreshold | Analysis of scale free topology for hard-thresholding. |
| pickHardThreshold.fromSimilarity | Analysis of scale free topology for hard-thresholding. |
| pickSoftThreshold | Analysis of scale free topology for soft-thresholding |
| pickSoftThreshold.fromSimilarity | Analysis of scale free topology for soft-thresholding |
| plotClusterTreeSamples | Annotated clustering dendrogram of microarray samples |
| plotColorUnderTree | Plot color rows in a given order, for example under a dendrogram |
| plotCor | Red and Green Color Image of Correlation Matrix |
| plotDendroAndColors | Dendrogram plot with color annotation of objects |
| plotEigengeneNetworks | Eigengene network plot |
| plotMat | Red and Green Color Image of Data Matrix |
| plotMEpairs | Pairwise scatterplots of eigengenes |
| plotModuleSignificance | Barplot of module significance |
| plotMultiHist | Plot multiple histograms in a single plot |
| plotNetworkHeatmap | Network heatmap plot |
| plotOrderedColors | Plot color rows in a given order, for example under a dendrogram |
| pmean | Parallel quantile, median, mean |
| pmean.fromList | Parallel quantile, median, mean |
| pmedian | Parallel quantile, median, mean |
| pminWhich.fromList | Parallel quantile, median, mean |
| populationMeansInAdmixture | Estimate the population-specific mean values in an admixed population. |
| pquantile | Parallel quantile, median, mean |
| pquantile.fromList | Parallel quantile, median, mean |
| prepComma | Prepend a comma to a non-empty string |
| prependZeros | Pad numbers with leading zeros to specified total width |
| preservationNetworkConnectivity | Network preservation calculations |
| projectiveKMeans | Projective K-means (pre-)clustering of expression data |
| proportionsInAdmixture | Estimate the proportion of pure populations in an admixed population based on marker expression values. |
| propVarExplained | Proportion of variance explained by eigengenes. |
| pruneAndMergeConsensusModules | Iterative pruning and merging of (hierarchical) consensus modules |
| pruneConsensusModules | Prune (hierarchical) consensus modules by removing genes with low eigengene-based intramodular connectivity |
| PWLists | Pathways with Corresponding Gene Markers - Compiled by Mike Palazzolo and Jim Wang from CHDI |

## -- Q --

|  |  |
| --- | --- |
| qvalue | Estimate the q-values for a given set of p-values |
| qvalue.restricted | qvalue convenience wrapper |

## -- R --

|  |  |
| --- | --- |
| randIndex | Rand index of two partitions |
| rankPvalue | Estimate the p-value for ranking consistently high (or low) on multiple lists |
| recutBlockwiseTrees | Repeat blockwise module detection from pre-calculated data |
| recutConsensusTrees | Repeat blockwise consensus module detection from pre-calculated data |
| redWhiteGreen | Red-white-green color sequence |
| reflectBranch | Select, swap, or reflect branches in a dendrogram. |
| relativeCorPredictionSuccess | Compare prediction success |
| removeGreyME | Removes the grey eigengene from a given collection of eigengenes. |
| removePrincipalComponents | Remove leading principal components from data |
| replaceMissing | Replace missing values with a constant. |
| returnGeneSetsAsList | Return pre-defined gene lists in several biomedical categories. |
| rgcolors.func | Red and Green Color Specification |
| rowQuantileC | Fast colunm- and row-wise quantile of a matrix. |

## -- S --

|  |  |
| --- | --- |
| sampledBlockwiseModules | Blockwise module identification in sampled data |
| sampledHierarchicalConsensusModules | Hierarchical consensus module identification in sampled data |
| scaleFreeFitIndex | Calculation of fitting statistics for evaluating scale free topology fit. |
| scaleFreePlot | Visual check of scale-free topology |
| SCsLists | Stem Cell-Related Genes with Corresponding Gene Markers |
| selectBranch | Select, swap, or reflect branches in a dendrogram. |
| selectFewestConsensusMissing | Select columns with the lowest consensus number of missing data |
| setCorrelationPreservation | Summary correlation preservation measure |
| shortenStrings | Shorten given character strings by truncating at a suitable separator. |
| sigmoidAdjacencyFunction | Sigmoid-type adacency function. |
| signedKME | Signed eigengene-based connectivity |
| signifNumeric | Round numeric columns to given significant digits. |
| signumAdjacencyFunction | Hard-thresholding adjacency function |
| simpleConsensusCalculation | Simple calculation of a single consenus |
| simpleHierarchicalConsensusCalculation | Simple hierarchical consensus calculation |
| simulateDatExpr | Simulation of expression data |
| simulateDatExpr5Modules | Simplified simulation of expression data |
| simulateEigengeneNetwork | Simulate eigengene network from a causal model |
| simulateModule | Simulate a gene co-expression module |
| simulateMultiExpr | Simulate multi-set expression data |
| simulateSmallLayer | Simulate small modules |
| sizeGrWindow | Opens a graphics window with specified dimensions |
| sizeRestrictedClusterMerge | Cluter merging with size restrictions |
| softConnectivity | Calculates connectivity of a weighted network. |
| softConnectivity.fromSimilarity | Calculates connectivity of a weighted network. |
| spaste | Space-less paste |
| standardColors | Colors this library uses for labeling modules. |
| standardScreeningBinaryTrait | Standard screening for binatry traits |
| standardScreeningCensoredTime | Standard Screening with regard to a Censored Time Variable |
| standardScreeningNumericTrait | Standard screening for numeric traits |
| stdErr | Standard error of the mean of a given vector. |
| stratifiedBarplot | Bar plots of data across two splitting parameters |
| subsetTOM | Topological overlap for a subset of a whole set of genes |
| swapTwoBranches | Select, swap, or reflect branches in a dendrogram. |

## -- T --

|  |  |
| --- | --- |
| TOMdist | Topological overlap matrix similarity and dissimilarity |
| TOMplot | Graphical representation of the Topological Overlap Matrix |
| TOMsimilarity | Topological overlap matrix similarity and dissimilarity |
| TOMsimilarityFromExpr | Topological overlap matrix |
| transposeBigData | Transpose a big matrix or data frame |
| TrueTrait | Estimate the true trait underlying a list of surrogate markers. |

## -- U --

|  |  |
| --- | --- |
| unsignedAdjacency | Calculation of unsigned adjacency |
| updateProgInd | Inline display of progress |
| userListEnrichment | Measure enrichment between inputted and user-defined lists |

## -- V --

|  |  |
| --- | --- |
| vectorizeMatrix | Turn a matrix into a vector of non-redundant components |
| vectorTOM | Topological overlap for a subset of the whole set of genes |
| verboseBarplot | Barplot with error bars, annotated by Kruskal-Wallis or ANOVA p-value |
| verboseBoxplot | Boxplot annotated by a Kruskal-Wallis p-value |
| verboseIplot | Scatterplot with density |
| verboseScatterplot | Scatterplot annotated by regression line and p-value |
| votingLinearPredictor | Voting linear predictor |

## -- W --

|  |  |
| --- | --- |
| WGCNAnThreads | Allow and disable multi-threading for certain WGCNA calculations |
